# Assessment of three medical and research laboratories using WHO AFRO_SLIPTA Quality Standards in Southwestern Uganda: a long way to go

**DOI:** 10.11604/pamj.2017.28.129.10995

**Published:** 2017-10-10

**Authors:** Ivan Mugisha Taremwa, Lucas Ampaire, Jacob Iramiot, Obed Muhwezi, Aloysius Matte, Herbert Itabangi, Hope Mbabazi, Jeninah Atwebembeire, Monicah Kamwine, Victoria Katawera, Yona Mbalibulha, Patrick Orikiriza, Yap Boum

**Affiliations:** 1 Faculty of Medicine, Mbarara University of Science and Technology, Mbarara, Uganda; 2 Epicentre Mbarara Research Centre, Mbarara, Uganda

**Keywords:** Quality, ISO 15189, SLIPTA, accreditation, laboratory assessment

## Abstract

**Introduction:**

While the laboratory represents more than 70% of clinical diagnosis and patient management, access to reliable and quality laboratory diagnostics in sub-Saharan Africa remains a challenge. To gain knowledge and suggest evidence based interventions towards laboratory improvement in Southwestern Uganda, we assessed the baseline laboratory quality standards in three medical and research laboratories in Southwestern Uganda.

**Methods:**

We conducted a cross sectional survey from October, 2013 to April, 2014. Selected laboratories, including one private research, one private for profit and one public laboratory, were assessed using the WHO AFRO_SLIPTA checklist and baseline scores were determined.

**Results:**

The three laboratories assessed met basic facility requirements, had trained personnel, and safety measures in place. Sample reception was properly designed and executed with a well designated chain of custody. All laboratories had sufficient equipment for the nature of work they were involved in. However, we found that standard operating procedures were incomplete in all three laboratories, lack of quality audit schemes by two laboratories and only one laboratory enrolled into external quality assurance schemes. The SLIPTA scores were one star for the research laboratory and no star for both the public and private-for-profit laboratories.

**Conclusion:**

While most of the laboratory systems were in place, the low scores obtained by the assessed laboratories reflect the need for improvement to reach standards of quality assured diagnostics in the region. Therefore, routine mentorship and regional supportive supervision are necessary to increase the quality of laboratory services.

## Introduction

The role of the laboratory in health care management remains instrumental [1]. It is estimated that 60-70% treatment and management decisions involve quantifiable laboratory data [2]. As the global risk of communicable and non-communicable diseases continues to upsurge, the role of laboratories towards clinical decisions including patient admissions, medications, and discharges; confirming diagnoses, conducting disease surveillance, and informing development of public health care policies has become more relevant [1-3]. Thus quality laboratory results are a cornerstone for better patient management and disease diagnosis [4]. The laboratory is a complex system that involves a recipe of activities and personnel. This complexion demands that procedures and processes be performed up to standard towards sustainable health care quality [5]. The World Health Organization (WHO) defines quality of health care as health care consisting of proper performance of interventions that are known to be safe, affordable, and have the ability to produce an impact on mortality and morbidity [6].

Despite the proven usefulness, laboratory quality standards (LQS) defined by improved accuracy, reliability and turnaround times remain at stake in sub-Saharan Africa (SSA), which is in part ascribed to the meager operational resources [7-9].Cognizant to this, the World Health Organization Africa regional office (WHOAFRO) designed a comprehensive approach of Stepwise Laboratory (Quality) Improvement Process Towards Accreditation (SLIPTA) to foster graduated laboratory quality performance [10]. SLIPTA is a framework for improving quality of public health laboratories in developing countries to achieve the requirements of the ISO 15189 standard [6]. The WHO AFRO SLIPTA program has been recommended for low resource set up [11]. The SLIPTA checklist is based on collection of patient samples, interpretation of test results, acceptable turnaround times, testing in medical emergency, routine internal quality control (IQC) and an 80%, 2-cycle pass rate on external quality control (EQC). This is augmented by 12 quality system essentials (QSE) hinged on documents and records; management reviews; organization and personnel; client management and customer service; equipment; internal audits; purchase and inventory; process control and internal & external quality assessment; information management; corrective action; occurrence/incident management and process improvement, and facilities and safety. Conformity to these is assessed based on scores of zero to a five-star SLIPTA grades, where 0 Star (0-142 points; <55%), 1 Star (143-165 points; <55-64%), 2 Stars (166-191 points; 65-74%), 3 Stars (192-217 points; 75-84%), 4 Stars (218-243 points; 85-94%) and 5 Stars (244-258; ≥95%) [12]. Although laboratory accreditation guarantees ability to perform to high quality standards [13], accredited laboratories in Africa remain scarce [8, 14]. The occurrence is not different for Uganda; a study done in Kampala indicated that only 4.7% of the assessed laboratories met the lowest score for accreditation under the WHO AFRO SLIPTA checklist [14]. Data from other regions of the country remains unknown; however, it seems obvious that their quality is deprived. This survey assessed the baseline laboratory quality standards of selected laboratories in Mbarara Municipality using WHO AFRO SLIPTA checklist.

## Methods

**Initiation of the survey:** This was an outcome of a study group on two master's degree programs in Medical Laboratory Science and Medical Microbiology offered in the faculty of medicine at Mbarara University of Science and Technology (MUST). The activity spun from the 10^th^ October, 2013 to 16^th^ April 2014. It involved three volunteer working teams (VWTs) and focal persons of the participatory laboratories (i.e. laboratory director/ coordinator/in-charge and quality managers). The VWTs comprised clinical laboratory professionals with experience of day-today laboratory activities and training on SLIPTA. The team had a series of lectures and tutorials led by a certified auditor registered with International Register of Certified Auditors (IRCA).

**Description of the assessed laboratories:** This assessment was done in Mbarara Municipality, located in the Mbarara district, south western Uganda. It is 266Km from Kampala capital city. The town serves as a conurbation of western region, and a gate way to Kigali, Bujumbura, Tanzania and Democratic Republic of Congo. Its population is estimated at 195,013 [15]. Mbarara Municipality caters for a multiplicity of patients from various ethnicities and towns in south and western regions. It has a major referral and teaching hospital (Mbarara Regional Referral Hospital), several specialized health facilities (public or private) and medical research facilities. The assessed laboratories were involved either in research or clinical diagnostics or both; and private for profit and public for profit. The diagnostic laboratories were stand-alone (defined as one that was not directly affiliated to a hospital or clinic), and by this design, clients/ samples were normally referred cases. In this survey, private for profit was defined as 'one owned by an individual or a group of individuals' and public for profit as 'one owned and/ or operated by a government institution'. The research laboratory was defined as a nonprofit one that supports laboratory tests done as part of research activities. The participating laboratories were chosen on the basis of work load, referral entity and research activities.

**Design of the survey tools used:** The WHO AFRO_SLIPTA checklist was used [12]. The checklist specifies requirements for quality and competency aimed to improve laboratory quality to established national standards. The elements of the checklist are based on ISO standard 15189: 2012 and, to a lesser extent, CLSI guideline QMS01-A4. The checklist comprised 110 questions worth 258 points. Responses to all questions had to be either, “Yes”, “Partial”, or “No”. Items marked “yes” received the corresponding point value (2, 3 or 5 points) depending on the relative importance of the quality essential and all elements of a question had to be present. Items marked “partial” received 1 point, and for those marked “no” were scored zero. A sum was obtained, and the obtained total was converted to a 0 to 5 star tiered accreditation score [12].

**Conduct of the survey :** Each of the three VWTs composed of 4 members were attached to a respective laboratory. A team began by scheduling for internal responsibilities to liaise with the respective focal persons. They prepared all the paper work that included the checklist, and they visited the laboratory as scheduled. The checklist was administered to focal persons, who were the quality managers, laboratory supervisors, human resource officers and designee. They documented the intricacy of the work done, staffing and baseline measures of quality and safety. The team adopted the SLIPTA laboratory audit guide (observations, reviews, asking open ended questions, talking to clients, specimen follow up through laboratory procedures) [4].

## Results

**Staffing levels of the participatory laboratories:** Participating laboratories had trained staff in laboratory medicine, with varying staff qualifications, ranging from certificate in medical laboratory techniques to master's degree, to meet work demands (Table 1). Also, they had support staffs like the driver, cashier, cleaner and data clerks.

**Table 1:** Showing the professional cadres that constituted the relevant staffing levels among participatory laboratories

Professional cadre	Number of personnel (staffs)			
Professional cadre	Research	Public for Profit	Private for profit	Total
Degree-holding	9	2	3	14
Diploma-holding	7	1	3	11
Certificate-holding	2	0	6	08
Cashier	-	1	1	02
Data Clerk	0	0	1	01
Cleaner	4	1	1	06
TOTAL	22	05	15	42

**Documents and records:** In all laboratories studied, documents were either missing or incomplete. Notably absent were the laboratory quality and safety manuals. Among the standard operating procedures (SOPs), SOPs for examination by referral laboratories, purchasing and inventory control, resolution of complaints and feedback, identification and control of non-conformities, corrective and preventive action, internal audits and equipment calibration were incomplete; while SOPs on specimen storage and retention were nonexistent.

**Management reviews:** Generally, all laboratories showed good management review with a work plan and budget. Only one laboratory had review of quality and technical records, and all laboratories had at least a system for communicating with the management about laboratory operations (obtained from meeting records).

**Organization and personnel:** In all three laboratories, no organogram was in place although there seemed to be a chain of command. Overall, complete work load schedules and coverage were present. Two laboratories neither had scheduled regular staff meetings nor personal filing system. In all the three laboratories, functional training policies and procedures for identification of training needs and plans were missing.

**Client management and customer service:** All participating laboratories lacked communication policy especially on delay to offer services. They also lacked evaluation tools and follow up on customer concerns as well as laboratory hand book for clients.

**Equipment:** Participating laboratories had equipments desired for the nature of work being done. However, there was poor adherence to equipment protocol as they all lacked labels and maintenance records. Overall, all laboratories lacked SOPs for equipment, date of purchase, validation, repair, disposal of obsolete equipment and failure contingency plan.

**Internal audits:** Two of the participating laboratories lacked internal audits. In the facility where this was done, we found out that although reports availed to responsible authorities for corrective/preventive actions, there was hardly any evidence in the subsequent audit reports.

**Purchasing and inventory:** There was 100% compliance to this, with service supplier performance reviews routinely done, up-to-date manufacturer/supplier list available, good budgetary projections, order tracking and a first-expire-first-out (FEFO) policy being practiced. This was enhanced by restricted access to the laboratory and more strict measures accessibility to the laboratory store.

**Process control, internal and external quality assessment:** Although specimens were labeled, packaged, stored appropriately, there was no procedural manual. All laboratories reportedly carried out internal quality assessment; however, very few records of these were available on site. Additionally, only one of the participatory laboratories were registered in an external proficiency testing scheme with an excellent score in most of the tests performed, however, all the assessed laboratories had a share of this. There was no written corrective action plan for failed proficiency panels.

**Corrective action:** Surprisingly, all laboratories had a poor system of corrective actions according to the SLIPTA requirements. There was no documentation on any laboratory corrective action regarding non-conformities, discordant results and troubleshooting of non-conformities.

**Occurrence management and process improvement:** This was generally poor. All the SLIPTA tools and documents like graphical tools and displays, quality indicators, outcome of external and internal audits for improvement of laboratory, checking of quality performance were lacking. Also, quality indicators (mainly turnaround time, rejected specimens and stock outs) were not selected, tracked and reviewed regularly.

**Facilities and safety:** All the participating laboratories were found to have adequate and well-furnished infrastructure requirements and adequate space for laboratory work. There was also separation of testing areas, and the laboratories had restricted access. Samples were separated from reagents. Additionally, work areas were clean, free of clutter and food stuffs. On the other hand, two of participatory laboratories lacked fire extinguishers, first aid kits and a designate safety officer.

**SLIPTA scores:** Two of the laboratories scored zero star; and only one laboratory scored a one star grade as indicated in Table 2.

**Table 2:** Showing the SLIPTA scores of the three laboratories

Laboratory identity	SLIPTA Score (Percentage)	Star grade (Interpretive range)
Private for profit	104 out of 258 (40.3)	ZERO (<55%)
Public for profit	83 out of 258 (32.2)	ZERO (<55%)
Research	160 out of 258 (62.0)	ONE (55-64%)

## Discussion

We found that the research laboratory (One-star) of the WHO AFRO SLIPTA checklist. This is a similar trend to findings of a survey conducted in Kampala, which reported that 4.7% of laboratories met the lowest quality standard (one-star) of the modified WHO-AFRO checklist [14]. Predisposition to the observed low quality is neither well studied nor understood; however, as earlier studies indicated, laboratory size and staff training may be some of the determinants [14, 16]. Although we did not study the effect of staffing and their training, the observed low scores for these laboratories concedes the fact that medical laboratory training may not suffice, but rather it ought to be augmented with more comprehensive quality management systems as earlier reported [11, 17]. Additionally, as the research laboratory scored the lowest acceptable accreditation level, it had clinical trials at the time of this survey, suggesting that this may have set certain quality needs. Besides, the authors are compelled to think of the relative affiliation of these laboratories and the possible funding that may impact on quality processes. Whereas the public for profit was affiliated a government university, it emerged that funding was a challenge. Similarly, despite many clients for the private for profit laboratory, it seems not to be sufficient to propel the fast demands of quality standards as higher costs may be incurred in running the facility. This finding is in agreement to what was earlier indicated that laboratories attached to Ministry of Health had higher workload and were better positioned to attract required funding to support laboratory quality performance [14]. Indeed private laboratories may not have sufficient funds and/or will to implement quality assurance process that may lead to extra cost in term of human resources, documentation and activities.

On the other hand, the research laboratory stood better funding opportunities and exposure, which may explain the relativeness in better quality performance. Moreover the running of clinical trials in consortium required working under Good Clinical and Laboratory Practice (GCLP) quality standards which surely impact in the quality of the laboratory. However this also highlights the differences in auditing process by SLIPTA as compared to clinical trial monitoring and audits. The assessment reveals that all participating laboratories showed deficiencies in virtually all the sections of WHO AFRO SLIPTA checklist. We found that key quality documents, internal and external quality controls, process improvement and quality controls were incomplete or non-existent yet they are critical to quality as earlier reported [18]. From this survey, it proves vital that mentorship of such facilities under the WHO AFRO SLIPTA quality standard would enhance their improvement towards accreditation.

## Conclusion

While most of the laboratory systems were in place, the low scores obtained by the assessed laboratories reflect the need for improvement to reach standards of quality assured diagnostics in the region. Although the research laboratory scored one star, there is room for improvement. Routine mentorship and supportive supervision are therefore necessary to increase the quality of laboratory services.

### What is known about this topic

- Laboratory quality is key in routine patient care and management;
- There are very few accredited laboratories in resource limited countries, which grossly hinders the quality of laboratory output;
- The WHO AFRO SLIPTA is a process that can be adopted by limited resources countries to improve on laboratory quality standards and work towards accreditation.

### What this study adds

- In our literature search, there was paucity of data regarding the SLIPTA quality status of laboratories in Uganda. To the best of our knowledge, this is the first survey to assess laboratory quality status based WHO AFRO SLIPTA checklist in Southwestern Uganda. It is hoped that this survey will hence serve as a baseline for other regions of Uganda, East Africa, Africa and other areas of the world;
- The survey used a standardized WHO AFRO SLIPTA checklist, therefore scores are depictive of international SLIPTA standards;
- Routine mentorship and supportive supervision are necessary to improve the laboratory quality standard based on SLIPTA requirements.

## Competing interests

The authors declare no competing interests.
